# *Bacillus amyloliquefaciens *GA1 as a source of potent antibiotics and other secondary metabolites for biocontrol of plant pathogens

**DOI:** 10.1186/1475-2859-8-63

**Published:** 2009-11-26

**Authors:** Anthony Arguelles-Arias, Marc Ongena, Badre Halimi, Yannick Lara, Alain Brans, Bernard Joris, Patrick Fickers

**Affiliations:** 1 Centre for Protein Engineering, Physiology and Bacterial Genetics, University of Liege, 4000 Liege, Belgium; 2 Walloon Centre for Industrial Biology, Bio-Industry Unit, Gembloux Agro-Bio Tech, University of Liege, B-5030 Gembloux, Belgium; 3 Walloon Centre for Industrial Biology, Microbial Technology Unit, University of Liege, 4000 Liege, Belgium; 4 Centre for Protein Engineering, Cyanobacteria Group, University of Liege, 4000 Liege, Belgium

## Abstract

**Background:**

Phytopathogenic fungi affecting crop and post-harvested vegetables are a major threat to food production and food storage. To face these drawbacks, producers have become increasingly dependent on agrochemicals. However, intensive use of these compounds has led to the emergence of pathogen resistance and severe negative environmental impacts. There are also a number of plant diseases for which chemical solutions are ineffective or non-existent as well as an increasing demand by consumers for pesticide-free food. Thus, biological control through the use of natural antagonistic microorganisms has emerged as a promising alternative to chemical pesticides for more rational and safe crop management.

**Results:**

The genome of the plant-associated *B. amyloliquefaciens *GA1 was sample sequenced. Several gene clusters involved in the synthesis of biocontrol agents were detected. Four gene clusters were shown to direct the synthesis of the cyclic lipopeptides surfactin, iturin A and fengycin as well as the iron-siderophore bacillibactin. Beside these non-ribosomaly synthetised peptides, three additional gene clusters directing the synthesis of the antibacterial polyketides macrolactin, bacillaene and difficidin were identified. Mass spectrometry analysis of culture supernatants led to the identification of these secondary metabolites, hence demonstrating that the corresponding biosynthetic gene clusters are functional in strain GA1. In addition, genes encoding enzymes involved in synthesis and export of the dipeptide antibiotic bacilysin were highlighted. However, only its chlorinated derivative, chlorotetaine, could be detected in culture supernatants. On the contrary, genes involved in ribosome-dependent synthesis of bacteriocin and other antibiotic peptides were not detected as compared to the reference strain *B. amyloliquefaciens *FZB42.

**Conclusion:**

The production of all of these antibiotic compounds highlights *B. amyloliquefaciens *GA1 as a good candidate for the development of biocontrol agents.

## Background

Phytopathogenic fungi affecting crop and post-harvested vegetables are a major threat to food production and food storage. Worldwide, this has led to important economic losses, particularly over the past few decades as agricultural production has intensified. Post-harvest food spoilage also represents a potential health hazard for humans due to the production by phytopathogens of toxic metabolites in the affected sites [1]. To face these drawbacks, producers have become increasingly dependent on agrochemicals. However, intensive use of these compounds has led to the emergence of pathogen resistance and severe negative environmental impacts. There are also a number of plant diseases for which chemical solutions are ineffective or non-existent as well as an increasing demand by consumers for pesticide-free food. Thus, biological control through the use of natural antagonistic microorganisms has emerged as a promising alternative to chemical pesticides for more rational and safe crop management. There is a large body of literature reporting the potential use of rhizosphere-associated bacteria in stimulating plant growth and biocontrol agents [2-4]. Among them, several strains belonging to the genus *Bacillus *and particularly to the *B. subtilis *and *B. amyloliquefaciens *species were reported effective for the biocontrol of multiple plant diseases caused by soilborne [5,6] or post-harvest pathogens [7-9]. Members of the *Bacillus *genus are thus among the beneficial bacteria mostly exploited as microbial biopesticides. *Bacillus*-based products represent about half of the commercially available bacterial biocontrol agents [10].

From a global viewpoint, the beneficial protective effect of these agents may rely on different mechanisms. By taking benefit from the nutrients secreted by the plant root, these bacteria efficiently colonise root systems and the surrounding soil layer (rhizosphere). In turn, they beneficially influence the plant through direct growth stimulation and/or by protecting it from infection by phytopathogens. Antibiosis through the production of antifungal metabolites and antibiotics is probably the best known and most important mechanism used by biocontrol bacteria to limit pathogen invasion in host plant tissues. Competition for iron traces in soils through siderophore production has also been postulated to be an important mechanism for the biocontrol activity of some rhizobacteria [11]. Another important mechanism relies on the ability of some strains to activate defence systems in the host plant. In other words, the beneficial bacterium can trigger a systemic resistance reaction that renders the host less susceptible to subsequent infection in distal tissues. This long-lasting phenomenon has been termed rhizobacteria-induced systemic resistance (ISR).

It is well known that some *Bacillus *species may synthesise numerous antimicrobial or, more generally, bioactive compounds with well-established activity *in vitro *[12]. However, except for a very limited number of strains [13,14], few studies that relate the global potential for antibiotic production with the biocontrol activity of a particular *Bacillus *strain have been reported. In this context, the *B. amyloliquefaciens *strain GA1 (formerly *B. subtilis *GA1, see below) displays high in vitro inhibitory activity toward growth of multiple fungal and oomycete plant pathogens [15]. When used as seed treatment, strain GA1 was shown to alleviate seedling diseases through direct antibiosis against soilborne pathogens (unpublished data). The strain was also shown to reduce post-harvest infection of apples caused by *B. cinerea*, the causative agent of grey mold disease [15]. These data suggest the secretion of multiple antibiotics and demonstrate the potential application of *B. amyloliquefaciens *GA1 as a biocontrol agent. In the present work, the genome of strain GA1 was sample sequenced to better characterise the genetic determinants directing the synthesis of antimicrobial metabolites that could be used in the field.

## Results

### Strain identification

**Figure 1:**
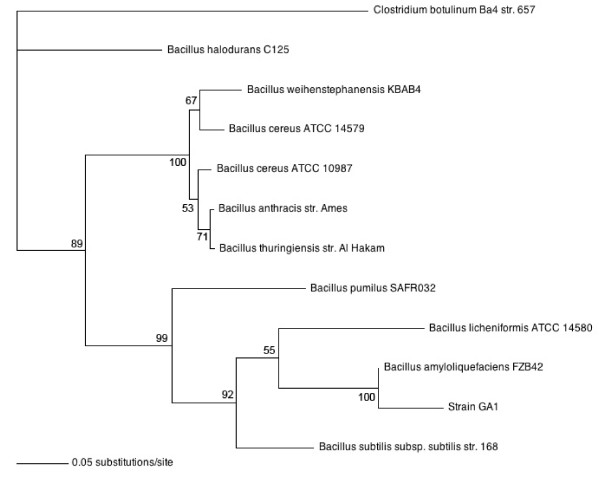
**Likelihood phylogenetic tree depicting the phylogenetic relationship between strain GA1 and other representatives of the *Bacillus *genera**: *B. amyloliquefaciens *FZB42 (NC_009725), *B. anthracis *str. Ames (NC_003997), *B. cereus *ATCC 10987 (NC_003909) *B. cereus *ATCC 14579 (NC_004722), *B. halodurans *C-125 (BA_000004), *B. licheniformis *ATCC 14580 (NC_006270), *B. pumilus *SAFR-032 (NC_009848), *B. subtilis subsp. subtilis *str. 168 (NC_000964), *B. thuringiensis *str. Al Hakam (NC_008600), *B. weihenstephanensis *KBAB4 (NC_010184). All sequences were aligned on *Clostridium botulinum *Ba4 str. 657 (NC_012658). Values for frequencies less than 50% are not given. The scale bars represent the number of substitutions per base position.

The *recN *and *recA *sequences from strain GA1 had 83% and 98% identity, respectively, with the sequence of *B. amyloliquefaciens *FZB42, while scores of 68% and 84% were obtained for *B. subtilis*. The phylogenetic tree (figure 1) constructed using cancatenated *recA *and *recN *sequences of related members of the *Bacillus *genus revealed that strain GA1 grouped with *B. amyloliquefaciens *FZB42 and is phylogenetically separated from *B. subtilis*. However, the phylogenetic tree suggests that the two strains are somewhat distant genetically.

### Genome analysis

**Figure 2:**
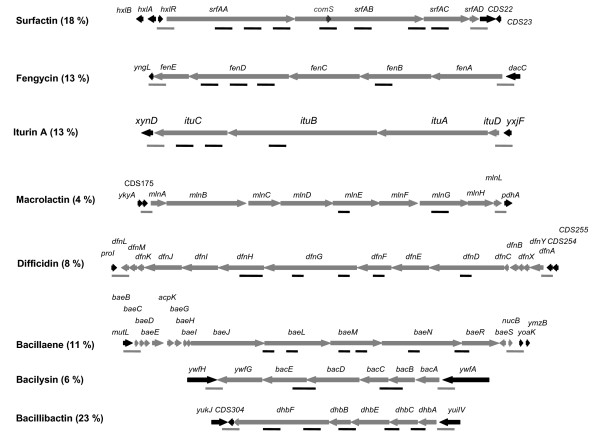
**Overview of the different gene fragments identified in strain GA1**. The grey arrows represent the different genes involved in the metabolite synthesis in *B. amyloliquefaciens *FZB42 while the black arrows represent the flanking genes. Black lines correspond to the gene fragments obtained during the sequencing of strain GA1, while grey lines indicate flanking regions amplified by PCR on strain GA1 genomic DNA with primer pairs described in Table 1. The values in parentheses give the percentage of the gene cluster determined in strain GA1.

Sequencing 500 clones from a shotgun library led to the determination of 461.5 kb of the *B. amyloliquefaciens *GAI chromosome and the identification of 358 protein-coding sequences. These presented, on average, 89% identity on amino acid level with that of *B. amyloliquefaciens *FZB42 and 76% with that of *B. subtilis*. Among these sequences, the partial ORFs of eight giant gene clusters directing the synthesis of bioactive peptides and polyketides by modularly organised mega-enzymes, the so-called non-ribosomal peptide synthetase (NRPS) and polyketide synthetase (PKS), were identified (figure 2). In addition, the *sfp *gene coding for a 4'-phosphopantetheinyl transferase responsible for the conversion of the apo-ACP domains of PKS and NRPS to their active holo-forms, was also detected in strain GA1 together with the regulatory gene *yczE *(data not shown).

**Table 1:** Metabolite production of *B. amyloliquefaciens *GA1 detected by HPLC-ESI mass spectrometry

Metabolite	Observed mass peak	Assignment	Reference
Surfactin	1030.8, 1046.8 [M+Na, K]+	C13-surfactin	[28]
	1044.8, 1060.8 [M+Na, K]+	C14-surfactin	[28]
	1058.8, 1074.8 [M+Na, K]+	C15-surfactin	[28]
			
Fengycin	1471.9, 1487.9 [M+Na, K]+	Ala-6 C15-fengycin	[28]
	1485.9, 1501.9 [M+Na, K]+	Ala-6 C16-fengycin	[28]
	1499.9, 1515.9 [M+Na, K]+	Ala-6 C17-fengycin	[28]
	1513.9, 1529.9 [M+Na, K]+	Val-6 C16-fengycin	[28]
	1527.8, 1543.8 [M+Na, K]+	Val-6 C17-fengycin	[28]
			
Iturin A	1066.1 [M+Na]+	C14-iturin A	[28]
	1079.7 [M+Na]+	C15-iturin A	[28]
			
Macrolactin	425.4 [M+Na]+	Macrolactin A[Bibr B28]	[19]
	511.4 [M+Na]+	7-o-malonyl macrolactin A	[19]
	525.4 [M+Na]+	7-o-succinyl macrolactin A	[19]
	629.3 [M+H-H2O]+	Macrolactin D	[19]
			
Difficidin	559.2 [M-H]-	Oxydifficidin	[18]
			
Bacillaene	583.5 [M+H]+	Bacillaene A	[18]
	605.5 [M+Na]+	Bacillaene B	[18]
			
Chlorotetaine	289.2 [M+H]+	Chlorotetaine (^35^Cl)	[20]
	291.1 [M+H]+	Chlorotetaine (^37^Cl)	[20]

**Figure 3:**
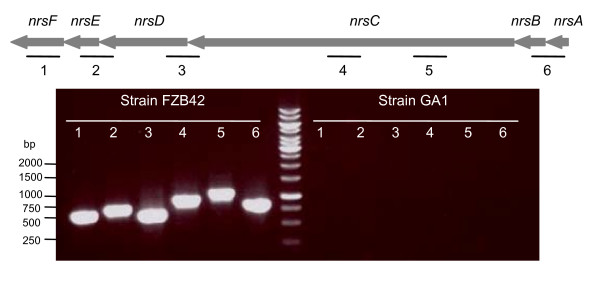
**Detection of the *nrs *operon in *B. amyloliquefaciens *strains FZB42 and GA1**. The grey arrows indicate the gene organisation in strain FZB42. The black lines represent the different gene fragments amplified by PCR using primer pairs as described in Table 1.

Fourteen gene fragments with homology toward gene clusters directing the synthesis of cyclic lipopeptide were obtained (figure 2). Of these, eleven had a high amino acid identity with *srf *(80-96%) or *fen *(41-92%) operon directing the synthesis of surfactin and fengycin in *B. amyloliquefaciens *FZB42. In strain GA1, the two gene clusters were found located in the same genetic environment as in strain FZB42 [13]; i.e. at the *hxlR*-*CDS22 *and *yngL-dacC *loci for *srf *and *fen *operon, respectively (figure 2). Similarly to *B. amyloliquefaciens *FZB42 and *B. subtilis *168, the *comS *gene, encoding a competence signal molecule, was found embedded within *srfAB *(figure 2). Three gene fragments directing the synthesis of an iturin lipopeptide were also detected. These fragments presented 48-82% amino acid identity with the *ituDABC *operon encoding the iturin A synthetase in *B. subtilis *RB14 [16]. In strain GA1, this operon was found located between the ORFs *xynD *and *yxjF *(figure 2). In addition, five gene fragments presented a high amino acid identity (87-93%) with the *dhb *gene directing the synthesis of the siderophore bacillibactin in *B. amyloliquefaciens *FZB42. They were found at an exactly conserved locus, i.e. between *CDS304 *and *yuilV *ORFs (figure 2). By contrast, the *nrs *operon directing the synthesis of a not yet identified NRPS peptide in *B. amyloliquefaciens *FZB42 [13] could not be detected by PCR techniques in strain GA1 (figure 3).

Several gene fragments involved in polyketide synthesis were also identified. Of these, thirteen presented a high amino acid identity with PKS genes directing the synthesis of the antibacterial compounds macrolactin (86-98%), difficidin (77-96%) and bacillaene (84-96%). In strain GA1, all these gene clusters were found to be collinear to that of *B. amyloliquefaciens *FZB42 [13]; i.e. between *CDS175 *and *pdhA *for macrolactin, *proI *and *CDS254 *for difficidin and *mutl *and *yoak *for bacillaene (figure 2).

**Figure 4:**
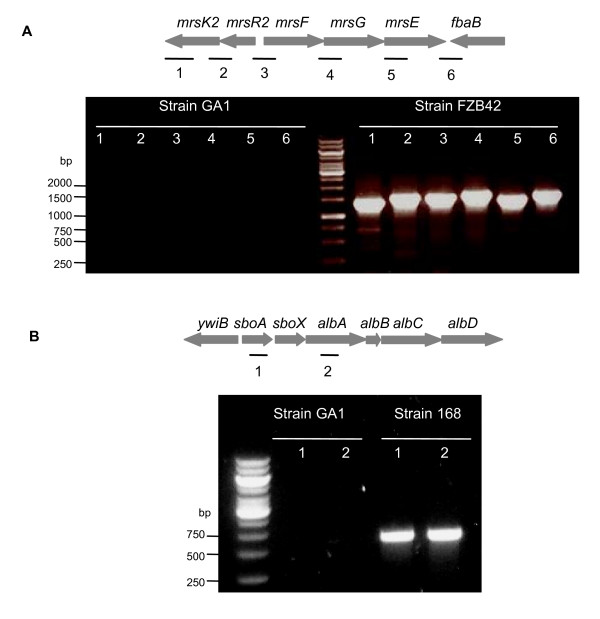
**Detection of the gene cluster involved in resistance toward bacteriocins in *B. amyloliquefaciens *strains FZB42 and GA1 **(A) and those responsible for subtilosin synthesis, immunity and transport in *B. subtilis *168 and *B. amyloliquefaciens *strains GA1. The grey arrows indicate the gene organisation in strain FZB42 (A) and 168 (B). The black lines represent the different gene fragments amplified by PCR using primer pairs as described in Table

In addition to these NRPS and PKS metabolites, genes involved in the synthesis of ribosomaly synthetised antibacterial compounds were detected in strain GA1. Indeed, their deduced amino acid sequence presented high identity (76-100%) with *bacB*, *bacC*, *bacD *and *bacE *genes encoding enzymes responsible for the synthesis and export of the di-peptide bacilysin, and were found located between ORFs *ywfH *and *ywfA *as in FZB42 strain. By contrast, none of the genes involved in *B. amyloliquefaciens *FZB42 in the resistance toward the bacteriocin mersacidin, nor those directing the biosynthesis of the cyclic bacteriocin subtilosin found in some strains of *B. amyloliquefaciens *could be detected by PCR techniques in strain GA1 (figure 4) [13,17].

### Analysis of the NRPS and PKS product of *B. amyloliquefaciens *GA1

Culture supernatants of *B. amyloliquefaciens *GA1 collected after 12 and 72 h of growth in Landy medium were concentrated by solid phase extraction and analysed by HPLC-ESI-MS. For samples collected after 72 h, three groups of mass peaks were detected (Table 1). Signals at *m/z *from 1030.8 to 1074.8 and from 1471.9 to 1543.8 were identified as surfactins and fengycins on the basis of data obtained both from the literature and from the analysis of pure compounds. For fengycins, signals at *m/z *of 1485.9 and 1513.9 and at 1499.9 and 1527.8 highlight an Ala/Val dimorphy at position 6 of the peptide ring for the C16 and C17 homologues, which is the characteristic trait of fengycin A and B. Mass peaks at *m/z *1066.1 and 1079.7 were found in accordance with the calculated mass values of sodium adducts of the C14 and C15 homologues of iturin A. By contrast, no peaks corresponding to the calculated mass of the various bacillomycin D homologues or to those of mycosubtilin could be detected under these conditions. This suggests that the iturins produced by strain GA1 correspond to the iturin A group. MALDI-TOF-MS analyses of crude supernatant were consistent with the mass patterns obtained by HPLC-ESI-MS (data not shown). From samples collected after 12 h of growth, signals at *m/z *425.4, 511.4, 525.4 and 629.3 were assigned to the molecular ions of the antimicrobial polyketide macrolactin A, 7-o-malonyl macrolactin A, 7-o-succinyl macrolactin A and macrolactin D respectively, whereas signals at *m/z *559.2, 583.5 and 605.5 were assigned to oxydifficidin, bacillaene A and bacillaene B (Table 1) based on data from the literature [18,19].

The production of siderophores by *B. amyloliquefaciens *GA1 was investigated by plating cells on CAS solid medium. After 72 h of incubation, the disappearance of the blue colour due to the ternary complex chrome azurol-S/iron (III)/hexadecyltrimethylammonium bromide in favour of a bright orange halo traduces the uptake of the ferric ions by the cells and thus the production of siderophore (i.e. bacillibactin) (data not shown).

### Production of antibacterial peptide

HPLC-ESI-MS analysis of freeze-dried samples of culture in Basic 77 medium gave peaks at m/z 289.2 and 291.1, which represents the typical mass spectrometric signature of the dipeptide antibiotic chloroteatine, a chlorinated derivative of bacilysin (Table 1) [20]. Surprisingly, no mass peak corresponding to this latter compound could be detected under our conditions. When samples were analysed by MALDI-TOF mass spectrometry, none of the bacteriocin subtilosin, mercacidin or subtilin mass signals could be detected (data not shown).

## Discussion

Recent taxonomic studies have revealed that *B. subtilis *is heterogeneous and should be considered as a group of closely related species [21]. In addition, *B. subtilis *and *B. amyloliquefaciens *are phenotypically similar and can be easily confused. Based on phenotypic and biochemical characterisations, strain GA1, initially isolated from strawberry cultures, was first assigned to *B. subtilis *(unpublished data, [15]). However, substantial molecular evidence suggested that the strain was more related to *B. amyloliquefaciens *than to *B. subtilis*. Thus, to accurately characterise the strain taxonomically, *recA *and *recN *genes, encoding DNA repair and recombination proteins, were sequenced and used to construct a phylogenetic tree. These two genes were selected because they had previously been shown to be effective in resolving closely related taxa [22]. The obtained phylogenetic tree clearly demonstrates that strain GA1 should be assigned to *B. amyloliquefaciens *rather than *B. subtilis*. The higher level of identity obtained for the 358 protein-coding sequence detected in strain GA1 to that of *B. amyloliquefaciens *FZB42 is in accordance with this result.

Plant-associated bacteria are known to play a key role in plant health by stimulating their growth and protecting them from phytopathogens, with this related to the production of a vast array of biologically active NRPS and PKS secondary metabolites. These metabolites have the same mode of synthesis, the so-called multicarrier thiotemplate mechanism, in which small monomer units, aminoacids and aryl acid in NRPS and acyl-CoAs in PKS are loaded, activated and condensed by mega-enzymes organised in iterative functional units [23]. The aim of this work was to better genetically characterise *B. amyloliquefaciens *GA1, with particular emphasis on gene clusters encoding these mega-enzymes. As they are encoded by large operons of 55 to 80 kb for PKS and 18 to 42 kb for NRPS, randomly sequencing 10% of the genome yielded enough data to point out the presence of these gene clusters in strain GA1.

Among NRPS antibiotics, *Bacillus amyloliquefaciens *GA1 was found to produce surfactin, iturin A, fengycin A and fengycin B. These are cyclic lipopeptides (CLP) composed of seven (surfactin and iturin A) or 10 α-amino acids (fengycins) linked to a β-amino (iturins) or β-hydroxy (surfactins and fengycins) fatty acid which may vary from C-13 to C-16 for surfactins, from C-14 to C-17 for iturins and from C-14 to C-18 for fengycins. CLPs have well-recognized potential biotechnology and biopharmaceutical applications due notably to their surface-active properties [24,25]. These surfactants may also play different roles in the development and survival of *Bacillus *strains in their natural habitat: increasing bioavailability of hydrophobic water-insoluble substrates, heavy metal binding, bacterial pathogenesis, quorum sensing, motility, biofilm formation etc. [26,27]. Other works have highlighted additional traits that are also very important for the fitness of *Bacillus *in the rhizosphere and for its efficacy as biocontrol agent [28].

The ability of *B. subtilis *to efficiently colonise surfaces of plant roots is a prerequisite for phytoprotection. This process relies on surface motility and efficient biofilm formation of the *Bacillus *cell populations that evolve and behave as structured and coordinated microcolonies adhering to root and on soil particle surfaces [29]. By modifying cell surface properties, surfactin and iturin were reported to positively influence cell spreading, swarming and biofilm formation [30-33] and thus may globally favour plant root colonisation. Furthermore, iturins and fengycins display strong antifugal activity and are inhibitory for the growth of a wide range of plant pathogens. Surfactins are not fungitoxic in themselves but retain some synergistic effect on the antifungal activity of iturin A [34]. In the context of biocontrol, the involvement of CLPs in direct antagonism of phytopathogens is thus obvious and was demonstrated by testing the pure compounds *in planta *or by correlating the biocontrol activity and use of non-producing or overproducing derivatives [5,35,36].

The role of fengycins produced by strain GA1 was demonstrated by the very effective disease control provided by treatment of fruits with CLP-enriched extracts and by *in situ *detection of fengycins in inhibitory amounts [15]. Another recently established role for lipopeptides from beneficial *Bacillus *isolates is the stimulation of the plant immune system [37]. Surfactins and, to a lesser extent, fengycins can induce a priming state in host plant which allows an accelerated activation of defense responses upon pathogen or insect attack, leading to an enhanced resistance to the attacker encountered [38]. Surfactins can be considered as a novel class of microbial-associated molecular patterns that can be specifically perceived by plant cells as signals to activate defense mechanisms [39]. The use of single strains evolving diverse mechanisms to reduce disease incidence is thus of prime interest. *Bacillus *isolates such as strain GA1 that co-produce the three CLP families should display such a multi-faceted biocontrol activity. That said, in strain GA1 the *itu *operon directing the synthesis of iturin A in *B. subtilis *RB14 [40] was surprisingly found inserted at exactly the same position as the expected bacylomycin D gene cluster from *B. amyloliquefaciens *FZB42 [32]. This suggests that an inter-species horizontal transfer of genes could have occurred between *B. subtilis *and *B. amyloliquefaciens*.

Besides lipopepides, a functional *dhb *gene cluster was shown in strain GA1 to direct the synthesis of the catecholic siderophore bacillibactin, a cyclic trimeric lactone of the 2,3-dihydroxybenzoyl-Gly-Thr monomer unit. Siderophores are high affinity ferric iron chelators that enhance the microbial acquisition of this element in environments where its bioavailability is extremely low, e.g. in soils. Thus, the presence of siderophore-producing microorganisms in the rhizosphere contributes to plant health by complexing iron and making it less available to phytophathogens that are generally not able to produce comparable Fe-transport systems [40].

In addition to peptides, polyketides are the other dominant family of secondary metabolites having revelant bioactivities. Similarly to *B. amyloliquefaciens *FZB42, three functional gene clusters directing the synthesis of difficidin, macrolactin and bacillaene were identified in strain GA1. Difficidin is an unsaturated 22-membered macrocylic polyene lactone phosphate ester [41] with broad spectrum antibacterial activity [42]. It inhibits protein biosynthesis [43] and was recently shown promising in its suppressive action against *Erwinia amylovara*, a devasting plant pathogen causing necrotrophic fire blight disease of apple, pear and other rosaceous plants [6]. By contrast, macrolactin and bacillaene have not yet been demonstrated to be directly related to biocontrol, although they are both antimicrobial agents that could be potentially useful in human medicine. Macrolactin, which consists of a 24-membered ring lactone, had the ability to inhibit murine melanoma cancer cells as well as mammalian herpes simplex viruses. It was also shown effective in protecting lymphoblast cells from HIV [44]. Similarly to difficidin, bacillaene is an inhibitor of prokaryotic protein synthesis constituted by an open-chain enamine acid with an extended polyene system. This compound displays antimicrobial activity toward human pathogens such as *Serratia marcescens*, *Klebsiella pneumoniae *and *Staphylococcus aureus *[45].

Bacilysin is a dipeptide composed of an L-alanine and the unusual amino acid L-anticapsin and represents one of the simplest peptide antibiotics known with antifungal and antibacterial activities [46]. L-anticapsin, released after uptake in susceptible cells, is an inhibitor of the glucose amine-6-phosphate synthetase, an enzyme essential in cell wall biosynthesis [47]. Due to its antibacterial activity, bacilysin is effective as a biocontrol agent, notably by inhibiting the growth of *E. amylovora*, the causative agent of fire blight disease [6]. It is also effective on the *Absidia ssp*., which is responsible for cutaneous and invasive zygomycosis in immunocompromised patients [48]. Besides bacilysin, some strains of *B. subtilis *also co-produced chlorotetaine, a chlorinated derivative of bacilysin with similar antibacterial activity [20,49]. Although no direct evidences are available, some experimental data suggest that the two compounds could share the same biosynthetic pathway [50]. Here, mass spectrometry analysis demonstrated that *B. amyloliquefaciens *GA1 synthetises only chlorotetaine as dipeptide antibiotic. While this behaviour is not clearly understood, this is to our knowledge the first *B. amyloliquefaciens *strain reported to produce chlorotetaine and the first strain to produce chlorotetaine and not bacilysin.

## Conclusion

In conclusion, the genetic and biochemical characterisation of the plant-associated *B. amyloliquefaciens *GAI demonstrated that the strain possesses a huge potential for biocontrol and plant growth promotion. Its natural competence and its possible genetic manipulation render strain GA1 attractive for further investigations for the development of green pesticides.

## Methods

### Strain indentification

Strain GA1 was identified by *recA *and *recN *sequence analysis [22]. Extraction and amplification of genomic DNA were performed as described elsewhere [51]. Fragments of *recA *and *recN *were amplified using primer pairs recAf/recAr and recNf/recNr, respectively (Table 2) and sequenced at the GIGA Genomics Facility (University of Liege, Liege, Belgium). Based on the 372 bp and 849 bp partial sequences of *recN *and *recA*, phylogenetically related bacteria were aligned using BLAST search against the GenBank data base. Multiple alignments with the *recN-recA *concatenated sequences of related species of the genus *Bacillus *were implemented using MUSCLE software [52]. A maximum likelihood phylogenetic tree was constructed by the parsinomy method using PAUP* software (version 4.0b8, [53]) and the sequence from *Clostridium botulinum *KBAB4 as outgroup. The parameters used for tree construction were as follows: (i) model of nucleotide substitution: GTR plus gamma distribution; (ii) number of substitution rate categories: 6; (iii) rate matrix parameter: estimated by program; (iv) number of bloodstrap replicates: 100.

**Table 2:** Oligonucleotides used in this study

Name	Sequence 5'-3'	Metabolite, gene or flanking region
bacAF1	GTGAAGGCCGTACTTTTGTCTGGC	Bacilysin, right
bacAR1	GGGGGGAAATACAGCTTCAGGGC	Bacilysin, right
beaBF1	GCCCGAAACGGCAGCGCCTG	Bacillaene, left
beaBR1	CGGAATGGAGGCTTTGATCCTCTG	Bacillaene, left
beaSF1	CGCAAAAGCTCTTCGACCGCCGTC	Bacillaene, right
beaSR1	CTCTCGTGCCGTCGGAATATCCGC	Bacillaene, right
dfnMF1	CGGAGTGAAACCGTGCCGGGATAAAGA	Difficidin, left
dfnMR1	GACCATTCAGAGCGGAAAGCTCC	Difficidin, left
dfnAF1	GGTGCGGCATGAAGATTTGAGATCACCG	Difficidin, right
dfnAR1	GGAGAGCACTTCAATTCCGACGTTGACC	Difficidin, right
dhbFF3	GCCTAGATGACATGGCGGCGG	Bacillibactin, left
dhbFR2	GCCGCCGTAGTCGTCCGTGAAGACCG	Bacillibactin, left
dhbAF1	CGCCTAAAGTAGCGCCGCCATCAACGC	Bacillibactin, right
dhbAR2	CCGCGATGGAGCGGGATTATCCG	Bacillibactin, right
fenAF1	CCTCGCTCCGCATGATCTTTTGG	Fengycin, left
fenAR1	CGGGAGCACGGTGGCAATGTG	Fengycin, left
fenEF1	GTTTCATGGCGGCGAGCACG	Fengycin, right
fenER1	GATTCGCGGGAAGCGGATTGAGC	Fengycin, right
ituF4	CTGCCTGCGTATATGATTCCGGC	Iturin A, left
ituR3	CCGTGATGATGCCGTTCTTCAATCC	Iturin A, left
ituF1	CGCCCGTGAAGGAGCAGCCG	Iturin A, right
ituR1	GCCAGGAAGCGGGGCTTCAC	Iturin A, right
mlnAF1	CGGCTGCGGGGGAAAAGATCCG	Macrolactin, left
mlnAR1	CAGCATCAGGGCGTGTATGACCTTC	Macrolactin, left
mlnIF1	GGAAGAAAAACAGTCGAGGCGATGCTG	Macrolactin, right
mlnIR1	GAGAAGCTCCGCCGTCACCAGTG	Macrolactin, right
srfAAF1	GCCCGTGAGCCGAATGGATAAG	Surfactin, left
srfAAR1	CCGTTTCAGGGACACAAGCTCCG	Surfactin, left
srfADF1	CCGTTCGCAGGAGGCTATTCC	Surfactin, right
srfADR1	CGCCCATCCTGCTGAAAAAGCG	Surfactin, right
ywfGF1	GAAGAGATCCTCGCCAAACATCCGG	Bacilysin, left
ywfGR1	GAGCGGATTGATCCCGCCGTC	Bacilysin, left
nrsAR1	GGAGGAGCTAATGACCCATCC	*nrsAB*
nrsBF2	CTCCTATGGAGCACGATCCAAC	*nrsAB*
nrsCF1	GGAATGCTGGTCCATTCAGCC	*nrsC*
nrsCR1	CAATCGCCAGTATCCTCGCAG	*nrsC*
nrsDF1	CCCAACTTATTTCACCGCCC	*nrsD*
nrsDR1	GTAAGGCTCGGCATTGAATCGAG	*nrsD*
nrsEF1	GGTGTGAAATCGTTGCGTTGG	*nrsED*
nrsER1	CAACAGGTAGCGTATGCGTGC	*nrsED*
nrsFF1	CGTACAGCCGGGCCAACTTCAAC	*nrsF*
nrsFR1	GGGCGTGCATATTAGGTGGAATC	*nrsF*
recAf	TGAGTGATCGTCAGGCAGCCTTAG	*recA*
recAr	TTCTTCATAAGAATACCACGAACCGC	*recA*
recNf	CTTTTGCGATCAGAAGGTGCGTATCCG	*recN*
recNr	GCCATTATAGAGGAACTGACGATTTC	*recN*
mrsF2	CTTGTGCCAATTCCCGGCTGAC	*mrsK2*
mrsR1	GGATGGCCGGTGTCTCACATG	*mrsK2*
mrsF3	CCATCGGTTTTCCCCATACCCATG	*mrsK2R2*
mrsR2	GTGGGGGGAGTTTTATGGCGGAG	*mrsK2R2*
mrsF4	GGTGAAGCCATCAGTGTCCGG	*mrsR2*
mrsR3	CCAGCACCATTCGGTCCAAGAAAACC	*mrsR2*
mrsF5	GTGGCTGTCTCAAACAGAACCGG	*mrsG*
mrsR4	GTTGCGGCTAATGGAAAACCCAGACC	*mrsG*
mrsF6	GGGCCTTTGTTAGGTGTATCCCTGG	*mrsE*
mrsR5	GGAAGACTCCCGCTTATGCCTAAC	*mrsE*
mrsF7	CCAGTGAACATGAGGAGCCC	*mrsE-fbabB*
mrsR6	CGCGATGACAAAAGAAGTCGCCG	*mrsE-fbabB*
sboA1	CTTCATTTGTTCCGCAATGTTCA	*sboA*
sboA2	CCCAGTGGGCCAATTGAATCCTCCC	*sboA*
ablB1	CGCGCAAGTAGTCGATTTCTAACA	*albA*
ablB2	CAAGTTTGGGCAAAAGAGCTTTTTC	*albA*

### Genome characterisation

For partial genome sequencing, a random shotgun approach was used. Total genomic DNA from strain GA1 was shared by nebulisation according to Surzycki [54]. DNA fragments of 2-3 kb in size were cloned into pMOSblue (GE Healthcare, Uppsala, Sweden) to establish a shotgun library. The inserts of 500 recombinant plasmids were sequenced from both ends. Sequences were processed with Vector NTI software (Invitrogen) and similarity search was performed using BLAST algorithm against the GenBank database. The chromosomic location of the different gene clusters identified in strain GAI was performed by sequencing their flanking regions using the FZB42 genomic sequence [13] as a template for PCR primer design (Table 2). The *nrsF*, *nrsE*, *nrsD*, *nrsC*, *nrsAB*, *mrsK2*, *mrsR2*, *mrsG*, *mrsE*, *fbaB*, *sboA *and *albA *gene fragments were amplified by PCR using the primer pairs listed in Table 1 and genomic DNA from *B. amyloliquefaciens *strains FZB42 and GA1 or from *B. subtilis *168 as a template.

### Identification of NRPS and PKS metabolites

For polyketide and lipopeptide production, *B. amyloliquefaciens *GAI was grown in Landy medium [55] at 37°C for 12 h and 72 h, respectively. Samples were extracted from the culture supernatant by solid phase extraction using Chromafix C18ec cartridge (Machery-Nagel, Duren, Germany). After binding and subsequent washing steps with MilliQ water (5 bed volume), metabolites were eluted with methanol (2 bed volume), dried under vacuum and resuspended in 100 μl of methanol. The resulting samples were analysed by reverse-phase high pressure liquid chromatography (Waters Alliance 2695/diode array detector) coupled with single quad mass spectrometer (Waters SQD mass analyser) on an X-Terra MS 150*2.1 mm, 3.5 μm column (Waters). Lipopeptides were eluted as described elsewhere [15] whereas polyketides were eluted in a binary solvent system (solvent A: water-0.1% formic acid, solvent B: acetonitrile-0.1% formic acid) as follows: 30% B for 5 min followed by a 5 min gradient from 30% B to 45% B and a subsequent 25 min gradient from 45% B to 100% B at a flow rate of 0.5 ml/min at 40°C. The identity of each metabolite was obtained on the basis of the mass of molecular ions detected in the SQD by setting electrospray ionisation (both positive and negative mode) conditions in the MS as source temperature, 150°C; desolvatation temperature, 325°C; nitrogen flow, 550 l/min; cone voltage 80 V. Matrix-assisted laser desorption ionisation time of flight (MALDI-TOF) mass spectrometry was performed on the crude culture supernatant on a Bruker Ultraflex TOF spectrometer (Bruker Daltonics) as described elsewhere [56]. Siderophore production was evaluated using the chrome-azurol-sulphonate (CAS) agar plate assay [57] using the growth medium described previously [58].

### Indentification of ribosomaly synthetised peptide

The ribosomaly synthetised peptide antibiotics were obtained by growing strain GA1 in Basic 77 medium [59] for 22 h. Peptides were extracted from freeze-dried culture filtrate with 30% acetonitrile-0.1% formic acid before being analysed by HPLC-ESI mass spectrometry. Molecule were eluted at a flow rate of 0,2 ml/min of a water/acetonitrile/formic acid mixture (87/12,9/0,1 v/v/v) and their identity was obtained on the basis of the mass of the molecular ions detected in the SQD by setting electrospray ionization (positive mode) condition in the MS as source temperature, 150°C; desolvatation temperature, 300°C; nitrogen flow, 550 l/min; cone voltage, 80 V. MALDI-TOF mass spectrometry was used in the reflectron mode of detection and with α-cyano-4-hydroxy-cinnamic acid as matrix.

## Competing interests

The authors declare that they have no competing interests.

## Authors' contributions

AA performed part of the genome characterisation and cultures for LC-MS analysis. MO performed the LC-MS analysis. BH constructed the shotgun library and annotated the sequence. AB and BJ planed and supervised part of the experiments. YL performed the phylogenetic analysis. PF wrote the final version of the manuscript and supervised the work. All authors approved the final version of the manuscript.
